# Subsocial Neotropical Doryphorini (Chrysomelidae, Chrysomelinae): new observations on behavior, host plants and systematics^1^

**DOI:** 10.3897/zookeys.332.5199

**Published:** 2013-09-19

**Authors:** Donald M. Windsor, Guillaume J. Dury, Fernando A. Frieiro-Costa, Susanne Lanckowsky, Jacques M. Pasteels

**Affiliations:** 1 Smithsonian Tropical Research Institute, Apartado 0843-00153, Balboa-Ancon, Panama, Republic of Panama; 2 McGill University, Department of Plant Science, Raymond Building, 21111 Lakeshore Road, Ste-Anne-de-Bellevue, Québec, H9X 3V9, Canada; 3 Centro Universitário de Lavras, Setor de Zoologia, Rua de Padre José Poggel, n°506, Centenário, CEP 37200-000, Lavras, Minas Gerais, Brazil; 4 Staatliches Museum für Naturkunde Karlsruhe, Referat Entomologie, Erbprinzenstr. 13, 76133 Karlsruhe, Deutschland; 5 Evolution Biologique et Ecologie, P.O. Box 160/12, Université libre de Bruxelles, 50 av. F.D. Roosevelt, B-1050, Brussels, Belgium

**Keywords:** Maternal care, Subsociality, Coleoptera, Chrysomelidae, Chrysomelinae, Neotropical

## Abstract

A summary of literature, documented observations and field studies finds evidence that mothers actively defend offspring in at least eight species and three genera of Neotropical Chrysomelinae associated with two host plant families. Reports on three *Doryphora* species reveal that all are oviparous and feed on vines in the Apocyanaceae. Mothers in the two subsocial species defend eggs and larvae by straddling, blocking access at the petiole and greeting potential predators with leaf-shaking and jerky advances. A less aggressive form of maternal care is found in two *Platyphora* and four *Proseicela* species associated with Solanaceae, shrubs and small trees. For these and other morphologically similar taxa associated with Solanaceae, genetic distances support morphology-based taxonomy at the species level, reveal one new species, but raise questions regarding boundaries separating genera. We urge continued study of these magnificent insects, their enemies and their defenses, both behavioral and chemical, especially in forests along the eastern versant of the Central and South American cordillera.

## Introduction

Arthropod parents influence the survival prospects of offspring in a multitude of ways. When parents bring resources to larvae, guide larvae to resources or actively shield offspring from predators and parasitoids, they are engaging in subsocial behavior (Alexander 1974, Wilson 1971, 1975). The study of subsocial behavior, additional to its intrinsic appeal, offers a perspective on selective forces at work during the earliest stages of insect social evolution, stages through which eusocial insects passed long ago. Understanding how environmental factors elevate the reproductive success of parents who defend offspring above those who abandon and direct investment toward future offspring remains a considerable challenge (Gilbert and Manica 2010, Tallamy and Wood 1986). This challenge is especially daunting for rare and diverse tropical beetles narrowly associated with rare host plants.

The Coleoptera include numerous examples of independently evolved subsocial behavior where the importance of competition, resource provisioning and defensive parental behavior can be examined (Costa 2006, Jordal et al. 2011). Within the leaf beetles, maternal care of offspring is found in just two of 15 subfamilies, the broad-shouldered leaf beetles (Chrysomelinae) and the tortoise beetles (Cassidinae), groups possibly more vulnerable to predators and parasitoids due to slow-moving and exposed immature stages (Jeffries and Lawton 1984, Cox 1994, Cornell and Hawkins 1995). Chrysomelinae larvae are often aposematic, aggregated (Santiago-Blay et al. 2012), chemically defended by eversible abdominal glands (Pasteels et al. 1994, Dobler et al. 2012) and, in some taxa, guarded by adults capable of secreting toxins obtained by sequestration of plant secondary metabolites (Pasteels et al. 2001, Termonia et al. 2002).

Below we review evidence of restricted host plant use and the presence of defensive maternal behavior in eight species of Neotropical Chrysomelinae. Detailed observations from one of these species suggests that mothers modify leaf resources in advance of bearing live offspring, and later block and herd movement of larvae among leaves on the same food plant. To clarify species relationships among Solanaceae-feeding species, some varying only in minor aspects of elytra color, we present genetic distance estimates obtained from mitochondrial sequence data for eleven described and one undescribed species, including both maternal care and non-care species.

## Methods

Tissue samples are preserved in ethanol at -80° C, and pinned adult voucher specimens are stored in the working collections of D.W., both at the Smithsonian Tropical Research Institute, Tupper Research and Conference Center, Panama City, Panama. Field observations reported below come from field notes and photographic records made at diverse occasions in Central and South America over the past 20 years.

### DNA extraction, sequencing and analysis

Adult Chrysomelinae were stored in 95% ethanol at -80° C, flight muscle removed and ground in 180 µl ATL tissue lysis buffer (Qiagen Inc., Valencia, CA USA) and 20 µl proteinase K with a sterile pestle, vortexed for 10 s and incubated overnight at 55° C. Following incubation, 200 µl AL lysis buffer (Qiagen Inc.) was added and the sample was heated at 70° C for 10 min, then 200 µl molecular grade ethanol was added to each sample. This mixture was then pipetted into a DNeasy mini spin column and centrifuged at 8000 rpm (~6000 *g*) for 1 min, then the flow-through and collection tube were discarded. The DNeasy mini spin column was placed in a new 2-ml collection tube and 500 µl wash buffer AW1 (Qiagen, Inc.) was added, the sample was centrifuged for 1 min at 8000 rpm, then the flow-through and collection tube were discarded. Again a new collection tube was used, 500 µl wash buffer AW2 (Qiagen, Inc.) was added and the sample centrifuged for 3 min at 14,000 rpm (20,000 *g*); the collection tube was then discarded. The mini column was placed in a 1.5-ml tube and 200 µl AE elution buffer (Qiagen, Inc.) was added, the sample was incubated for 2.5 min at room temperature, and the sample centrifuged for 1 min at 8000 rpm (~6000 *g*). Extractions were held at -20° C between use, and at -80° C for long-term storage. The primers: C1-J-1718F (26-mer; 5’-GGA GGA TTT GGA AAT TGA TTA GTT CC-3’) and C1-N-2191 (26-mer; CCC GGT AAA ATT AAA ATA TAA ACT TC-3’) (Simon et al. 1994) were used to amplify the mitochondrial cytochrome oxidase 1 gene (COI) in a volume of 20 µl: 1 µl DNA sample, 2 µl 10x buffer (Applied Biosystems Inc., Foster City, CA, USA), 2 µl MgCl_2_ (25 µM), 1µl nucleotide mix (8 mM each), 0.8 µl dimethyl sulfoxide 5%, 1 µl each primer (20 mM), 0.2 U *Taq* DNA polymerase (AmpliTaq, Applied Biosystems Inc.) plus sterile water. The PCR cycling conditions were: 94° C for 2 min, 10 cycles of 94° C for 30 s, 46° C for 30 min, 72° C for 45 min, then 24 cycles of 94° C for 30 s, 48° C for 30 min, 72° C for 45 min, and finally 72° C for 10 min and 10° C for 2 min.

Forward and Reverse sequences were combined and reconciled in Sequencher v5 (Gene Codes Corporation, Ann Arbor, MI, USA) and trimmed, leaving a single 472 bp fragment, which was then translated to amino acids and found free of stop codons. Sequences from ten species were combined with three sequences from GenBank creating an ingroup of 12 species (20 individuals) and a single outgroup species. Where possible we included two separate individuals from the same population. New sequences were deposited in GenBank under accession numbers in Table 1. Evolutionary relationships of the samples were inferred by Bayesian analysis, with 2 million generations, and Maximum Likelihood analysis, with 100 bootstrap pseudo-replications. The ideal partitioning strategy and models of nucleotide substitution were determined using PartitionFinder v.1.0.1 (Lanfear et al. 2012) and this scheme was implemented in both analyses. The strategy was determined with three character sets, one for each codon position of COI. The partitioning scheme divided the dataset in two partitions: Partition (1): first and second codon positions of COI; Partition (2): third codon positions of COI. Pairwise genetic distance estimates were calculated in MEGA version 5.0 (Tamura et al. 2011) using the Kimura 2-parameter model.

**Table 1.** Apocyanaceae and Solanaceae feeding taxa mentioned in the text, collection and host plant information, life history characteristics, accession numbers and references.

**Chrysomelinae species**	**Location**	**Host Plant Family^1^**	**Host Plant Species**	**Larval Group Defense**	**Maternal Care**	**Reproduction**	**Genbank Accession Numbers**	**References**
*Doryphora paykulli* (Stål, 1859)	Gamboa, Panama Province, Panama	Apo	*Prestonia seemannii* Miers (subfamily Apocynoideae)	aggregated	yes	oviparous	-	new observation
*Doryphora reticulata* (Fabricius, 1787)	Boqueirão Reserve, Minas Gerais State, Brazil	Apo	*Prestonia tomentosa* R. Br. (subfamily Apocynoideae)	aggregated	yes	oviparous	-	new observation
*Doryphora* sp. near *Doryphora punctatissima* (Olivier, 1790)	El Porvenir, Meta Province, Colombia	Apo	*Prestonia isthmica* Woodson (subfamily Apocynoideae)	aggregated	no	oviparous	-	Eberhard 1981
*Eugonycha melanostoma* (Stål, 1859)	Serra do Japi, Jundiaí, São Paulo State, Brazil	Sol	*Solanum* sp.	aggregated	no	larviparous	-	Vasconcellos-Neto and Jolivet 1994
*Platyphora amabilis* (Baly, 1859)	Yasuní, Orellana Province, Ecuador	Sol	*Solanum* sp.	?	?	?	AY055517	new observation
*Platyphora anastomozans* (Perty, 1832)	Serra do Japi, Jundiaí, São Paulo State, Brazil	Sol	*Solanum bullatum* Vell., *Solanum muritianum* (Scopoli), *Solanum sancta-catarine* Dunal, *Solanum megalochiton* Mart., *Solanum rufescens* Sendt.	aggregated	no	larviparous	KF251110, KF251111	Vasconcellos-Neto and Jolivet 1994
*Platyphora aulica* (Olivier, 1807)	Montagne de Kaw, Roura Commune, French Guiana	Sol	*Solanum rugosum* Dunal, *Solanum torvum* Sw.	solitary	no	larviparous	KF251112, KF251113	new observation
*Platyphora conviva* (Stål, 1858)	Serra do Japi, Jundiaí, São Paulo State, Brazil	Sol	*Solanum* spp.	aggregated^2^^,^^3^	no	larviparous	-	Medeiros and Vasconcellos-Neto 1994, Vasconcellos-Neto and Jolivet 1994
*Platyphora fasciatomaculata* (Stål, 1857)	Ijuí, Rio Grande do Sul State, Brazil	Sol	*Solanum mauritianum*	aggregated	no	larviparous	-	Medeiros et al. 1996
*Platyphora microspina* Bechyné, 1954	Cerro Campana, Panama Province	Sol	*Markea megalandra* (Dunal)	aggregated	yes	larviparous	KF251120	new observation
*Platyphora nigronotata* (Stål, 1857)	Serra do Japi, Jundiaí, São Paulo State, Brazil	Sol	*Solanum bullatum*, *Solanum muritianum*, *Solanum sancta-catarine*, *Solanum megalochiton*	aggregated	no	larviparous	KF251121	Medeiros 1991
*Platyphora nitidissima* (Stål, 1857)	Serra do Japi, Jundiaí, São Paulo State, Brazil	Sol	*Solanum bullatum*	aggregated	no	larviparous	-	Medeiros and Vasconcellos-Neto 1994, Vasconcellos-Neto and Jolivet 1994
*Platyphora quadrisignata* (Germar, 1824)	Serra do Japi, Jundiaí, São Paulo State, Brazil	Sol	*Solanum variabile* Mart., *Solanum fastigatum* Willd	aggregated^2^	no	larviparous	-	Medeiros and Vasconcellos-Neto 1994, Vasconcellos-Neto and Jolivet 1994, Medeiros et al. 1996
*Platyphora selva* Daccordi, 1994	La Selva Biological Station, Heredia Province, Costa Rica	Sol	*Solanum* (Witheringia) *heteroclita*.	aggregated	yes	larviparous	-	Choe 1989
*Platyphora sphaerica* (Jacoby, 1903)	Serra de Baturite, Fortaleza, Brazil	Sol	*Solanaceae*, 2 spp.	solitary	no	larviparous	AY055529	Termonia et al. 2002
*Platyphora vinula* (Stål, 1858)	Ijuí, Rio Grande do Sul State, Brazil	Sol	*Solanum* sp. aff. *Solanum megalochiton*, *Solanum sancta-catarinae*	aggregated	no	larviparous	-	Medeiros et al. 1996
*Proseicela antennalis* (Kirsch, 1883)	Reventador, Napo Province, Ecuador	Sol	*Solanum* sp.	?	?	?	KF251114, KF251115	new observation
*Proseicela bicruciata* Jacoby, 1880	Yanayacu Biological Station, Napo Province, Ecuador	Sol	*Solanum abitaguense* S. Knapp	aggregated	yes	larviparous	KF251116, KF251117	new observation
*Proseicela crucigera* (Sahlberg, 1823)	Serra do Japi, Jundiaí, São Paulo State, Brazil	Sol	*Solanum decompositiflorum* Sendtn.	aggregated	no	larviparous	-	Medeiros 1991, Medeiros and Vasconcellos-Neto 1994)
*Proseicela flavipennis* (Erichson, 1847)	Reventador, Napo Province, Ecuador	Sol	*Solanum abitaguense* S. Knapp	?	?	?	KF251118, KF251119	new observation
*Proseicela* sp. n. “Yasuni”	Yasuní, Orellana Province, Ecuador	Sol	*Cuatresia* sp.	aggregated	yes	larviparous	KF251126, KF251127	new observation
*Proseicela spectabilis* (Baly, 1858)	Río Malo & Reventador, Napo Province, Ecuador	Sol	*Solanum abitaguense* S. Knapp, *Solanum* sp. (sect. Dulcamara)	aggregated	yes	larviparous	KF251122, KF251123	new observation
*Proseicela vittata* (Fabricius, 1781)	Montagne de Kaw, Roura Commune, French Guiana	Sol	*Solanum morii* S. Knapp	aggregated	yes	larviparous	KF251124, KF251125	new observation
*Stilodes modesta* Jacoby, 1882	Cerro Campana, Panama Province, Panama	Mal	*Banisteriopsis* sp.	aggregated	no	oviparous	AY055522	new observation

^1^ Apo=Apocynaceae, Ast=Asteraceae, Sol=Solanaceae, Mal=Malpighiaceae

^2^=nocturnally active

^3^=larvae cut and cover themselves with trichomes

## Results

### Field observations of behavior and natural history

#### Doryphora paykulli

(Stål, 1859)

http://species-id.net/wiki/Doryphora_paykulli

##### Remarks.

**Figure 1.**
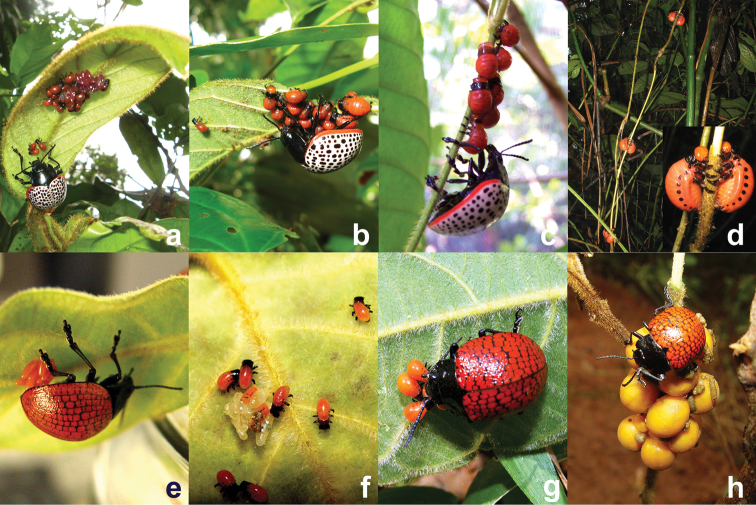
Maternal care providing *Doryphora* species, **a** *Doryphora paykulli* female with eggs and first instar larvae under an apical leaf of *Prestonia seemanii* (photo by S.L.) **b** female straddling a mix of first and second instar larvae (photo by S.L.) **c** *Doryphora paykulli* larvae moving to a new leaf followed by their mother (photo by S. Van Bael) **d** *Doryphora paykulli* larvae stripping the cortex of their host while descending in pairs to pupate, (photo by D.W.) **e** *Doryphora reticulata* ovipositing under apical leaf of *Prestonia tomentosa* in Central Brazil (photo by F.F.) **f** *Doryphora reticulata* larvae on the natal leaf (photo by F.F.) **g** female *Doryphora reticulata* stradding first instar larvae (photo by F.F.) **h** *Doryphora reticulata* female tending fully-developed larvae at the base of the food plant just prior to pupating underground (photo by F.F.).

According to Blackwelder (1982) the range of this large beetle (19.8 ± 1.5 mm, n=6) (Figs 1a–c) extends from Mexico to Nicaragua. However, specimens collected later (L.D. Gomez near San Vito, Costa Rica; H. Stockwell, Cerro Campana, Panama Prv., 30 V 70 and 18 VII 76; D.M.W., Los Santos Prv., Cerro Canajagua, 25 V 92 and Colon Prv., Cerro Galera, 1 V 02; M. Cuignet, Colon Prv. Sta. Rita Ridge Rd. km 2, 2 XI 02; S. Van Bael, Bocas del Toro Prv., Chiriqui Gde., 17 I 04; M. Membache, 1 VI 92, Colon Prv., Gamboa; S. Lankowski, Panama Prv., Parque Metropolitano, 15 IV 07) indicate the species extends to at least 10 km east of the Panama Canal. These records plus observations of a *Doryphora paykulli* adult following a tightly arranged group of larvae moving between leaves on their food plant near Chiriquí Grande, Bocas del Toro Province (S. Van Bael, pers. comm.) documents the presence of the species in the Caribbean as well as in the Pacific lowlands of Panama and provides the first unequivocal record of subsocial habits for the species.

Subsequently, individual *Doryphora paykulli* adults were observed (D.W., S.L.) during late April and early May of 2005 moving slowly and feeding among low vegetation. Pairs of individuals were observed interacting aggressively on the small leaf fragments remaining on the host plant, *Prestonia seemannii* Miers (Apocynaceae) under late dry season conditions along trails in the Parque Metropolitano (elevation 30 m, 8°59.24'N, 79°32.797'W), Panama City. Whether these were contests over resources or precopulatory courtship is unclear, however, analysis of video taken of one of these interactions shows the use of the mesosternal horn in dislodging a competitor, much as described by Eberhard (1981) for the closely related beetle, *Doryphora* sp. near *punctatissima*. Adult females were discovered during mid-May of both 2005 and 2007 in close proximity to eggs attached to the underside of cupped, newly expanded *Prestonia seemannii* leaves (Fig. 1a) on the western slope of Cerro Pelado, Gamboa (elevation 95 m, 9°7.29'N, 79°41.78'W). Eggs measured 3.5 × 0.9 mm and at first were an opaque, cherry-red, with the chorion becoming transparent and larvae distinguishable as development progressed. Clutches initially contained ten eggs with roughly ten new eggs added each day over the course of 3 to 5 days. The female became noticeably more defensive the second day of oviposition, straddling the eggs and jerking from side to side apparently in response to movement by the observer.

Larval emergence began on days 6 and 7 following first oviposition with clutches (n=3) at that time containing from 40–50 eggs. Within 24 hours after larvae began to emerge many of the original eggs were missing, apparently consumed by early-emerging larvae such that clutches were reduced to 8, 15 and 20 surviving larvae accompanied by some intact, opaque, less-developed eggs and opened eggs with red-colored residues of once-developing larvae visible under close inspection (Fig. 1). One day later an adult was observed (S.L.) returning to a natal group after having fallen from the plant on a detached leaf, then deposit a single egg which was immediately consumed by a nearby larva. First instar larvae (1.5–2.0 mm in length) had small black heads at the time of emergence, these easily distinguished them from second instar larvae appearing, 1 to 2 days later with red head capsules and a larger and more rotund appearance. Larvae expanded rapidly in size following their first meals but did not feed on leaf tissue until after the first molt, 2 to 3 days after emerging from the egg. Mothers at times tightly straddled their aggregated first and second instar larvae on the natal leaf, preventing their advance down the leaf petiole (Fig. 1b). While guarding, mothers reacted aggressively by charging to the edge of the leaf when a thin stick was introduced to the area by an observer. Charges, stamping and shaking continued for at least two minutes after the stimulus was presented and removed. The strongest reaction was given to a camera held approximately 10 cm under and to the side of the natal leaf. The mother seemed to be reacting to the camera lens–suggesting that a mirror held near guarding mothers might provide a non-invasive means of assessing defensiveness. On one occasion an *Ectatomma tuberculatum* (Olivier) worker was observed to pass by the base of the petiole, eliciting aggressive shaking of the natal leaf and short charges, after which the ant reversed course and departed that portion of the plant. As larvae became larger and began moving between leaves the intensity of the mothers’ reactions to foreign stimuli appeared to subside. Increasingly, mothers were seen feeding on leaves and leaf petioles, rather than guarding, as larval development proceeded.

Once the natal leaf was consumed, larvae began moving down the petiole to the stem, where they then moved either up or down in smaller groups to other leaves, sometimes moving as solitary individuals. Mothers, often fed from the pedicel of the leaf just consumed, occasionally accompanied by one or two larvae. Mothers actively trampled upon the backs of larvae still located on the pedicel, in effect pushing them away from the leaf and toward the stem. Mothers on other occasions stepped on and over larvae, rapidly tapping larvae with antennae and tarsi until they reversed direction. After leaving the natal leaf, mothers resumed guarding one of the several larval groups that reassembled. However, some groups continued to split into ever smaller units and moved to adjoining leaves and stems, leaving mothers guarding smaller sets of offspring and spending more time travelling among groups in what seemed to the observer as an effort to herd offspring back together (Fig. 1c). Mothers also increasingly divided their time between guarding and feeding on the cortex of the stem, girdling the vine over distances from a few cm to nearly 1 m. Seven days following eclosion larvae were arranged largely in doublets, girdling stems as intact leaves had disappeared in substantial sections of the plant. Larvae then descended in unguarded pairs to pupation sites by backing down the stem from which all cortex was stripped (Fig. 1d), effectively killing that section of the plant. By day eleven, most larvae had descended the host plant and moved along small above-ground roots into the leaf litter. One mother was last seen guarding two slow-developing larvae high (2 m) on the plant thirteen days after oviposition. Development from first oviposition to larvae wandering on the ground took approximately 20 days. Several larvae collected and placed in a plastic container with moist leaves, molted at day 4 and eclosed as teneral adults on days 18 and 19, a metamorphosis period slightly shorter than the 24 days estimated for *Doryphora* sp. near *punctatissima* by Eberhard (1981). Development from oviposition to the eclosion of adults in mid-June required 35 days. Monthly visits to the study area throughout the rest of the year were successful in finding solitary, feeding adults on nearby host plants but not in finding signs of additional reproductive activity. Thus, this species seems to have but a single generation per year timed to the period of accelerated leaf growth by its host plant. The nearly synchronous May onset of reproduction in both *Doryphora paykulli* in Panama and *Doryphora* sp. near *punctatissima* in Colombia (Eberhard 1981) is likely the product of similar climate regimes in the two species’ ranges and subsequent effects on host phenology. Finally, the high morphological similarity of these neighboring species, the presence of larval cannibalism in both, but the presence of maternal care in only one, raises intriguing questions regarding the lability of defensive behaviors and underscores the importance of reconstructing phylogenetic relationships for as many *Doryphora* species as possible.

#### Doryphora reticulata

Fabricius, 1787

http://species-id.net/wiki/Doryphora_reticulata

##### Remarks.

Recent observations by F.F. reveal clearly that maternal care is expressed by *Doryphora (Megistomela) reticulata* (Fabr.) in the cerrado of south-central Brazil (Fig. 5) (see also photo in Chaboo 2011). Photographs of this species in the Boqueirão Biological Reserve, Minas Gerais of Brazil (elevation 1200 m; 21°20.76'S, 44°59.49'W) in 2005 clearly show behaviors strikingly similar to that observed in *Doryphora paykulli* in Panama. Females oviposit on the underside of partially-expanded, apical leaves of *Prestonia tomentosa* (Apocynaceae) (Fig. 1e, f). Larvae emerge and are tightly straddle-guarded by the female (Fig. 1g), but unlike *Doryphora paykulli* and *Doryphora* sp. near *punctatissima*, no larval cannibalism of eggs was observed. Larvae guarded by the mother continued to feed on leaves and strip cortex, eventually descending to the ground tended by the mother prior to pupating nearby in the soil (Fig. 1h). Indeed, of the many Chrysomelinae species associated with Solanaceae and other plant families studied at Serra do Japi and other sites near Campinas in Central Brazil (Table 1), *Doryphora reticulata* is the only species in which mothers are known to actively guard their larval brood. Inferences regarding subsocial habits in *Platyphora conviva* (Reid et al. 2009) are incorrect according to J. Vasconcellos-Neto (personal communication, 2013). Further, ongoing studies in the eastern lowlands of Bolivia by one of the authors (D.W.) have found no evidence of maternal care occurring in any of 16 species of Doryphorini.

#### Platyphora selva

Daccordi, 1994

http://species-id.net/wiki/Platyphora_selva

##### Remarks.

Within New World Chrysomelinae, reports of subsociality until recently were limited to a single species studied at the La Selva Field station in the Atlantic lowlands of Costa Rica (Choe 1989). However a misidentification of that species (not by the author) lead to erroneous attribution of subsocial behavior to *Labidomera suturalis*, rather than to an unidentified species of *Platyphora*. The species was subsequently described and named *Platyphora selva* by Daccordi without comments on Choe’s behavioral observations (Daccordi 1993). As noted by Reid et al. (2009), this first record of subsocial behavior in Neotropical chrysomelines led to a number of reports citing the original paper and repeating the taxonomic error (e.g. Windsor and Choe 1994, Kudô and Hasegawa 2003, Costa 2006).

Choe (1989) observed 18 guarding *Platyphora selva* females in two different years, all feeding on *Lycianthes (Witheringia) heteroclita* Sendtm. (Solanaceae) in the Atlantic lowlands of Costa Rica. His observations were remarkable in first describing how females of this species tightly guarded offspring by straddling. By removing mothers from roughly half of the families, he was able to demonstrate that guarding was highly effective in preventing predation by the gigantic ponerine ant, *Paraponera clavata* Fab. The importance of maternal defenses in reducing losses to parasitoids, however, was not investigated. Further, it was noted that mothers always guarded groups of four or fewer larvae; but eggs of the beetle were never observed during the study. From observations of related taxa (see below) we now suspect that *Platyphora selva* is not oviparous, but instead deposits temporally isolated clutches of four larvae. This inference remains to be documented and is based on the habits of the morphologically similar species, *Platyphora microspina*, which occurs widely (but rarely) in neighboring Panama. Regrettably, sequence data are not yet available for *Platyphora selva*.

#### Platyphora microspina

(Bechyně, 1954)

http://species-id.net/wiki/Platyphora_microspina

##### Remarks.

**Figure 2.**
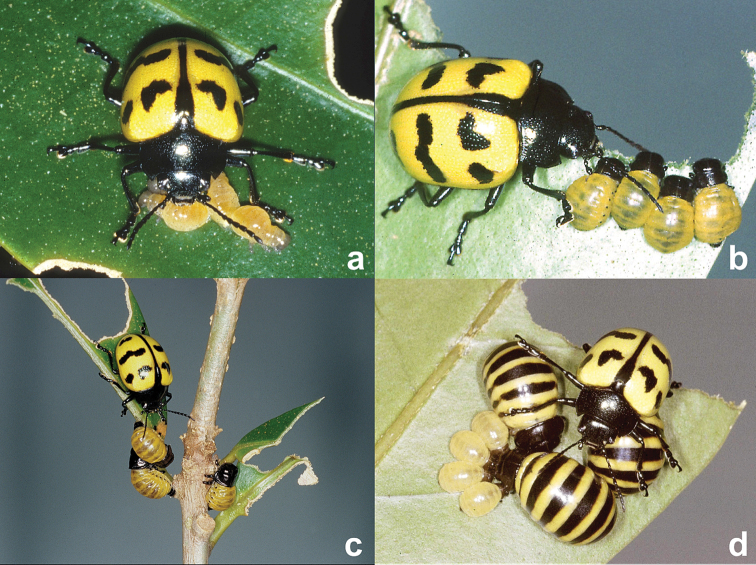
Maternal care providing *Platyphora microspina* in Panama, **a** female with recently deposited larvae (photo by D.W.) **b** female guarding mid-sized larvae (photo by D.W.) **c** female and young larval brood moving among leaves (photo by D.W.) **d** female tending overlapping cohorts of larvae (photo by D.W.).

*Platyphora microspina* was initially observed on Cerro Campana (Parque Nacional Altos de Campana), along a ridgeline approximately 50 m west and up-slope from the Podocarpus trail in July 1999 (elevation 900 m; 8°41.07'N, 79°55.82'W). Large numbers of adult and immature beetles were observed feeding on *Markea megalandra* (Dunal), a woody hemiepiphyte which grows within the canopy of forests at elevations of 1000–2000 m in Western and Central Panama (Correa et al. 2004). Larvae and adults of *Platyphora microspina* (Fig. 2a) were largely associated with quick-growing sprouts coming from a portion of the plant damaged earlier by limb fall. A small number of *Platyphora microspina* including one female tending three small and partially-sclerotized larvae were moved to a terrarium in an air conditioned laboratory containing host plant cuttings to facilitate observations. Larvae remained physically in contact with one another, often beneath one or more legs of the mother during the first three days. However, as larvae grew in size and spent more time feeding, the mother moved to the side of the group for the remaining 10–12 days of development and feeding (Fig. 2b). Single larvae occasionally left the aggregation, apparently to find new leaves, and through alternative bouts of substrate tapping with the tip of the abdomen—approximately one to two taps per second for two to three minutes—isolated larvae appeared able to call or stimulate their siblings and mother to visit new feeding sites (Fig. 2c). The mother was also observed physically nudging inactive larvae. As the first cohort neared the end of its feeding period the mother deposited another cohort of 4 larvae, briefly leaving 7 larvae of two distinct cohorts and size classes together under the mothers care (Fig. 2d). One day later larvae in the first cohort fell to the base of the terrarium, became inert and later pupated. An additional two cohorts produced by the same mother, each containing 4 larvae, were subsequently observed in the lab. Larvipositions occurred over a span of 28 days, 10 days between cohort 2 and 3, 17 days between cohorts 3 and 4. The larval feeding period for the second cohort lasted 21 days. The non-feeding prepupal period lasted 8 days and the pupal period 7 days. Thus, the interval between larviposition and adult emergence takes approximately 5 weeks in this species. Observations were terminated after the fourth cohort, so reproduction possibly continues for an even longer period in this relatively non-seasonal, premontane forest. The species is dependent upon the continued presence of its hemiepiphytic host plant on Cerro Campana and similar small refuges along the cordillera passing through Panama. Approximately eight years after these observations were made a single adult specimen resembling *Platyphora microspina* was collected at the Cana field station near the Colombian border. One year later at the same site, a group of recently emerged adults of the same species were observed on a woody shrub in the family Solanaceae, 3–5 m to the side of the entrance of to Cana gold mine. Preliminary analysis of its COI gene sequence shows that it is nearly identical to that of *Platyphora microspina* on Cerro Campana. Continuing observations at this and similar remote sites, coupled with molecular sequencing, should add considerably to our knowledge of this species and its relationship to *Platyphora selva* and similar species in South America.

#### Proseicela vittata

(Fabricius, 1781)

http://species-id.net/wiki/Proseicela_vittata

##### Remarks.

**Figure 3.**
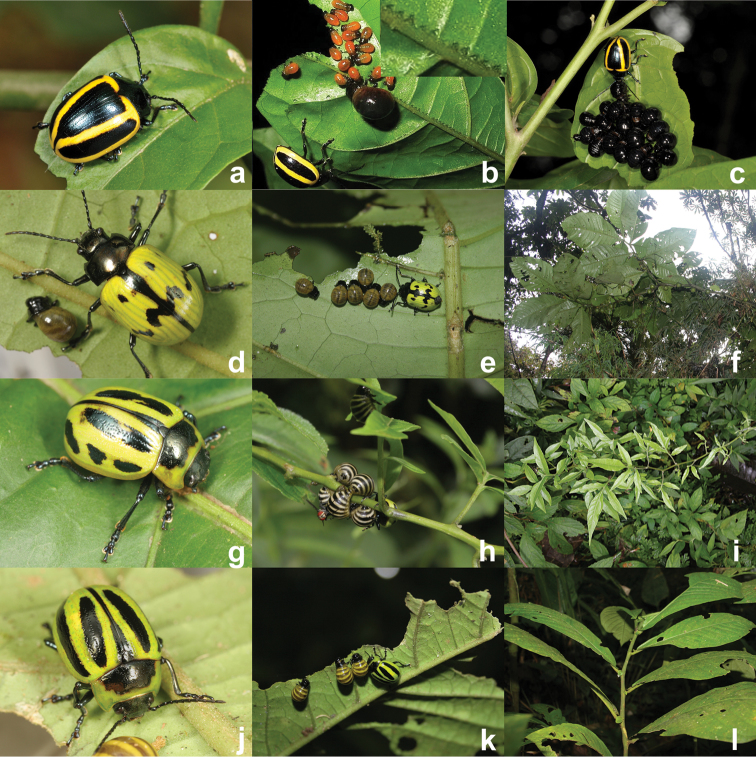
Maternal care providing *Proseicela* species, **a** *Proseicela vittata* adult (Photo by D.W.) **b** *Proseicela vittata* female and larvae from two cohorts. Insert shows detail of vein pinching along approximately 1cm of the primary vein (Photo by D.W.) **c** *Proseicela vittata* female with late stage larvae (Photo by D.W.) **d** *Proseicela bicruciata* adult female, (photo by G.D.) **e** *Proseicela bicruciata* female tending larvae (photo by G.D.) **f** *Proseicela bicruciata* food plant, *Solanum abitaguense* (photo by G.D.) **g** *Proseicela spectabilis* adult (photo by G.D.) **h** *Proseicela spectabilis* with nearly full-grown larval brood and tachinid parasitoid (photo by G.D.) **i**. *Proseicela spectabilis* host plant, Solanum sp. (photo by G.D.) **j** *Proseicela* sp. n. adult female (photo by G.D.) **k** the same female tending three feeding larvae feeding on *Cuatresia* sp. (Solanaceae) (photo by G.D.) **l** wider view of the host plant (photo by G.D.).

Female *Proseicela vittata* (Fig. 3a) were observed by D.W. tending offspring during each of three visits to Montagne de Kaw, French Guiana (elevation 150 m; 4°32.686'N, 52°09.151'W), 11–18 April 2010, 25–31 January 2011 and 19 June–2 July 2012. All individuals and family groups were found on a single species of host plant, *Solanum morii* S. Knapp, a 1-3 m shrub with glabrous leaves and pendulous green and white fruit and a colonist of disturbed areas (Plate 121a, Mori et al. 2002). We searched host plants for this species mainly along logging roads. While numerous individuals and family groups were found on each trip, most groups contained older larvae. Only four females were found tending recently deposited larvae, 11 to 18 in number, which had not begun to feed. The brood tended by one female contained a single large larva feeding and resting beside 17 freshly deposited larvae (Fig. 3b). Within 2 days the single large larva descended alone to pupate, a sign that while broods may overlap in this species (as in *Platyphora microspina*) the period of overlap is brief. Normally, individual larvae within cohorts were remarkably similar in size (Fig. 3c). The only exception came if they were observed on day 2 or 3 while molting was in progress. Despite three observation periods per day of approximately 15 min per family, possible predators and parasitoids were rarely observed. And while a *Pachycondyla* ant or a carabid beetle may have been responsible for the abrupt loss of 15 of 17 larvae from one female over night, ongoing predation has yet to be observed in this species.

The 17 larvae belonging to another female were observed to take approximately 30 hours to consume the entire lamina of the natal leaf. While the last of the leaf was being consumed some larvae began to molt while still on the remnants of the natal leaf. The mother maintained a tight grip on the leaf petiole (blocking behavior), but eventually larvae pushed by and began traversing nearby stem and petioles solitarily or in small groups. Commonly families split into two or more separated feeding groups at this stage, with the mother usually remaining with a larger group. Groups often reunited but others remained separated until pupation. The transition from the natal to second leaf appears to be a crucial and dynamic time for larvae and events proceeded differently for most groups. During this period mothers moved actively among different leaves and branches in what seemed to be attempts to herd and reconstitute a single larval group. While we observed what we interpret as herding behavior in most species in this report, its possible importance to group safety and success remains open and in need of experimental study.

#### Proseicela bicruciata

Jacoby, 1880

http://species-id.net/wiki/Proseicela_bicruciata

##### Remarks.

Strong indications of maternal care in *Proseicela bicruciata* (Fig. 3d) were gathered by G.D. while walking trails in the montane cloud forest of the Yanayacu Biological Station and Center for Creative Studies, Napo Province, Ecuador (2150 m elevation, 0°36.27'S, 77°53.25'W) during the first week of July 2011. A total of five females were found, each tending small groups of uniform larvae (Fig. 3e), on the undersides of large leaves of *Solanum abitaguense* S. Knapp growing in streamside habitats (Fig. 3f). The group containing the smallest larvae was composed of nine individuals, while groups with larger larvae contained five and six individuals. The group with five larvae had two individuals separated on leaves 20–30 cm in different directions from the central three larvae arranged in a small rosette with heads to the inside and the mother to the side.

#### Proseicela spectabilis

(Baly, 1858)

http://species-id.net/wiki/Proseicela_spectabilis

##### Remarks.

Observations of *Proseicela spectabilis* were taken by G.D. while walking the main trail leading to the Cascadas de San Rafael, Reventador, Napo province, Ecuador (1300 m elevation; 0°6.07'S, 77°35.18'W) on July 17, 2011. A single female (Fig. 3g), was perched half above its larvae and half on the branch of its food plant. Six of the seven larvae in the formation were tightly aggregated around the thin stem of the plant, while the seventh larva was on a leaf a few centimeters away. A tachinid fly was present on the dorsal surface of a larva located on the side opposite the mother (Fig. 3h). The fly departed when the observer approached but promptly returned to land on the larva opposite the mother. The host plant (Fig. 3i) was later identified as a nightshade, *Solanum* sp. section *Dulcamara*.

### *Proseicela* sp. n. “Yasuni”

**Remarks.** A single *Proseicela* adult tending a group of three larvae (Fig. 3j, k) was observed and photographed by G.D. while walking a trail leading to the 50 ha forest dynamics plot, within the Estación Científica Yasuní (ECY), Orellana province, Ecuador (220 m elevation; 0°40.83'S, 76°23.89'W) on 15 July 2011. Following discovery the larvae formed a small rosette with heads to the inside. The female and larvae were attached to the underside of a leaf of *Cuatresia* sp. (Solanaceae) (Fig. 3l). Two other adults of the same species were found nearby, one on a different branch of the same host and the other on an unidentified plant. According to M. Daccordi, this is an undescribed species.

### Observations on other Solanaceae-feeding species

**Figure 4.**
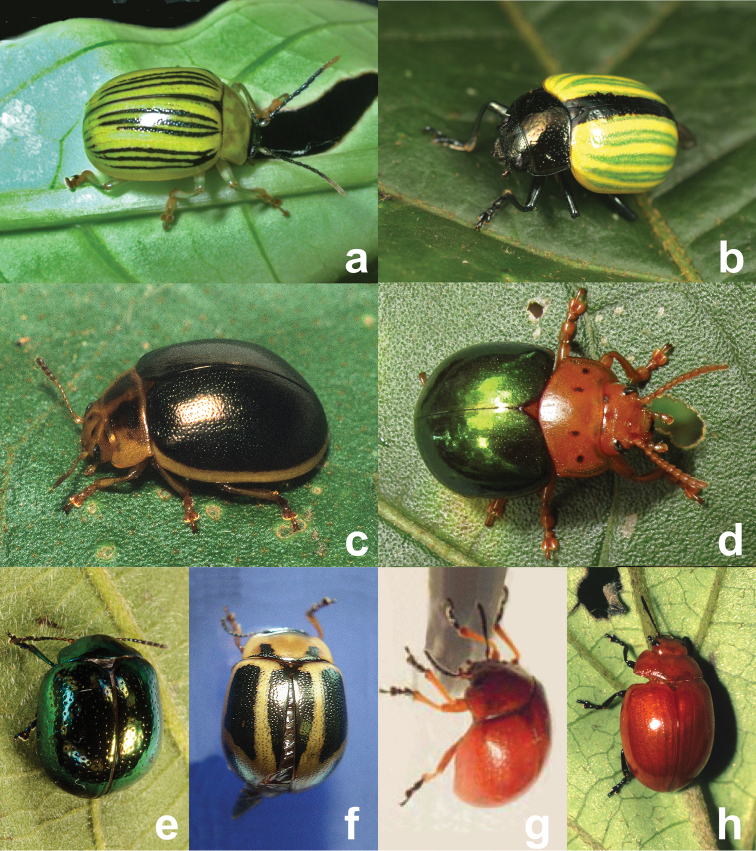
Other Solanaceae associated Chrysomelinae of unknown habits (**a, b, c, g**), known not to provide maternal care (**d, e, f**) and outgroup taxon (**h**), **a** *Proseicela antennalis* (Photo by D.W.) **b** *Proseicela flavipennis* (Photo by G.D.) **c** *Platyphora amabilis* (Photo by D.W.) **d** *Platyphora aulica* (Photo by D.W.) **e** *Platyphora nigronotata* (Photo by D.W.) **f** *Platyphora anastomozans* (Photo by D.W.) **g** *Platyphora sphaerica* (Photo by J.P.) **h** *Stilodes modesta* (Photo by D.W.).

Two additional *Proseicela* species are known from understory Solanaceae at Cascadas de San Rafael, Ecuador. Several *Proseicela antennalis* adults (Fig. 4a) were collected by D.W. and J.P. from unidentified Solanaceae, 12 August 2001. Additionally, one *Proseicela flavipennis* adult (Fig. 4b) was collected by G.D. at the same site on leaves of *Lycianthes glandulosa* (Ruiz & Pav.) Bitter, 17 July 2011. As none of the individuals in these two species were reproducing, their interactions with offspring remain unknown, however high morphological similarity to adults of other *Proseicela* species in the area suggest they are good candidates to be subsocial. *Platyphora amabilis* (Fig. 4c) adults were observed and collected from a well-armed solanaceous food plant growing in open, roadside habitats at the Estación Científica Yasuní (Fig. 4d) by D.W. during August 2001. Larvae of this species were not observed. *Platyphora aulica* (Fig. 4e) was observed on numerous occasions and collected from *Solanum rugosum* Dunal and *Solanum torvum* Sw. in the same roadside habitats and the same dates in French Guiana as *Proseicela vittata*. An adult female *Platyphora aulica* placed in a container with abundant food, deposited approximately one larva per day. Mothers of this species walk away from their live born larvae, leaving all to feed and develop as solitary individuals. Three additional Solanaceae-feeding species from Brazil are included in the analysis that follows. *Platyphora anastomozans* (Perty) and *Platyphora nigronotata* (Stal) specimens were collected from Serra do Japi, Brazil where aspects of their biology has been studied (Medeiros and Vasconcellos-Neto 1994, Vasconcellos-Neto and Jolivet 1994). *Platyphora sphaerica* Jacoby specimens were observed on several unidentified solanaceous food plant species near Fortaleza by J.P., 3 April 1999.

### Species validity and life history characteristics of Solanaceae-feeding taxa

**Figure 5.**
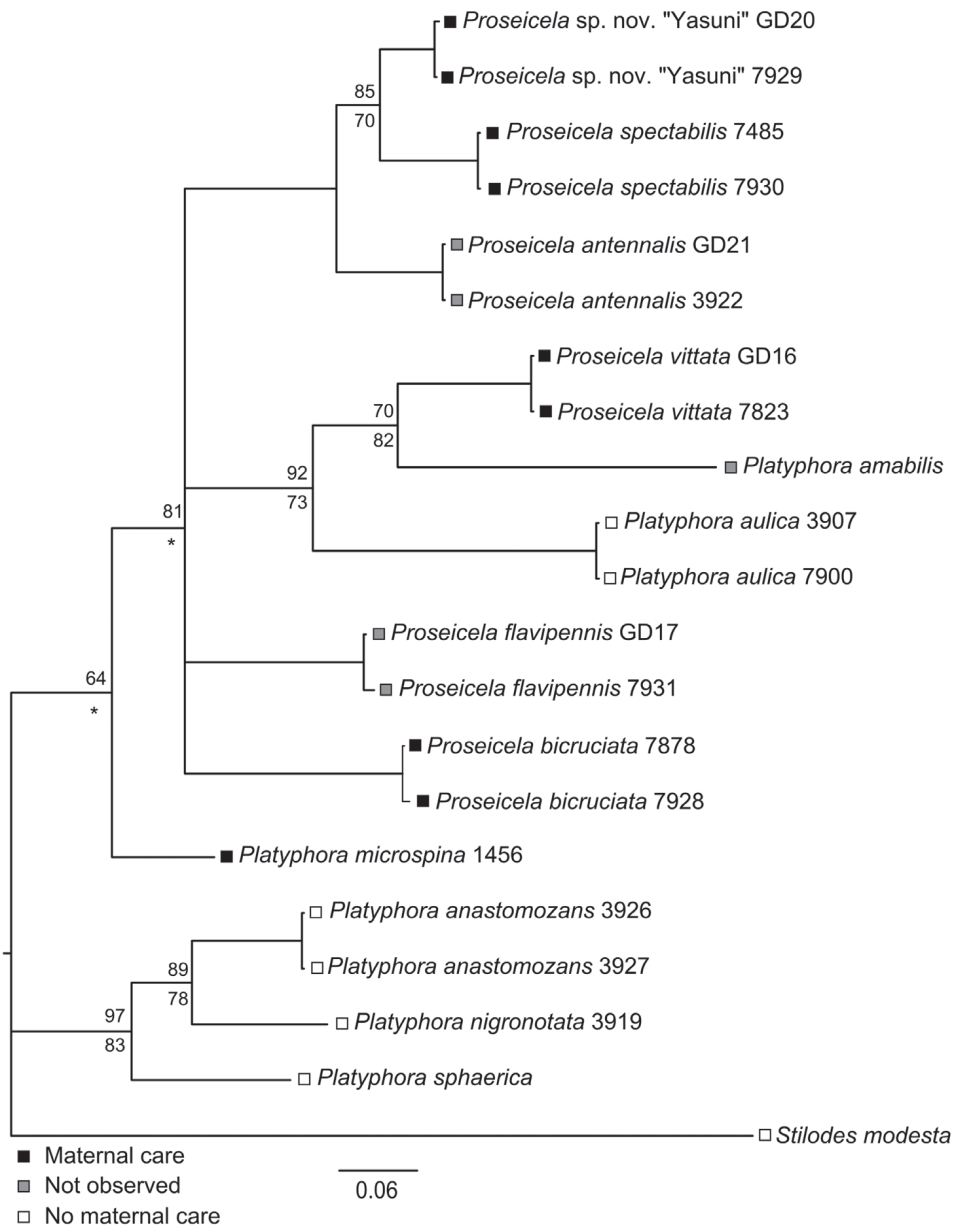
Bayesian Consensus tree of 472 bp COI sequences obtained for 12 species of Central and South American Solanaceae-feeding Doryphorini and one outgroup. For nodes with less than 100% support, Bayesian values are placed above node, Maximum Likelihood bootstrap values below the node, while asterisks (*) indicate nodes with different taxon placement under ML analysis and thus are not strictly comparable.

Two lines of evidence support the validity of the *Solanum*-feeding species recognized above. The first is the expert opinion of M. Daccordi who has reviewed each of the species in this report including voucher specimens and finds only one unidentified species, *Proseicela* sp. n. “Yasuni”. Nevertheless, while an experienced taxonomist can separate species, the criteria can be subtle and based on few characters. The five Ecuadorean *Proseicela* species differ only in subtle aspects of elytral and pronotal color pattern and for this reason we sought genetic evidence of species limits. The topology of the resulting consensus tree generated by Bayesian Inference (Fig. 5) resolved all taxa as separate entities, with Bayesian support values ranging from 64 to 100%, while Maximum Likelihood bootstrap estimates for nodes common to both trees ranged from 70 to 100%. The smallest pair-wise genetic distance estimates occurred between the pairs, *Proseicela* sp. n. “Yasuni”, *Proseicela spectabilis* (6.4%) and *Proseicela antennalis* (7.5%), and between *Proseicela spectabilis* and *Proseicela antennalis* (9.0%) with all remaining pair-wise distances ranging between 10.2 and 18.6%. Thus all mean distances fall above the 2 to 6% range considered a threshold for distinct species, depending on COI evolution rates within particular clades (Hajibabaei et al. 2006, Wiemers and Fiedler 2007). While the polytomy in our tree does obscure relationships among some *Proseicela* species and clades, the remaining placement of taxa raises questions regarding generic assignments, especially those for *Platyphora amabilis* and *Platyphora aulica*, the latter seemingly a species which has secondarily lost subsocial habits common among closest taxa.

Aspects of the biology of 20 Neotropical species of Solanaceae-feeding Doryphorini (i.e. excluding *Leptinotarsa* and similar genera) are now known and where the mode of reproduction has been recorded, all (17) are live-bearing or “larviparous” (Table 1). Further, 15 species are reported to have aggregated larvae, while only two species (*Platyphora aulica* and *Platyphora sphaerica*) have solitary larvae. Maternal care is now known to occur in six species and known not to occur in another eleven species. While five *Proseicela* species are maternal care providers, *Proseicela crucigera* in south-central Brazil has gregarious larvae but mothers do not extend care after larviposition (João Vasconcellos-Neto, personal communication, 2013).

## Discussion

Because of the caveats to which mitochondrial data are subject (Rubinoff et al. 2006), and limitations in taxon sampling, the relationships of the Apocynaceae and Solanaceae associated taxa to one another and to other Doryphorini associated with eight other host plant families is best left to a multi-gene analysis (Dury et al. in preparation). However, the COI data are sufficient to confirm the validity of 12 Solanaceae feeding species and supports the existence of two major clades, one of which contains non-maternal care providing species distributed primarily in the “cerrado” of central and southern Brazil. The second clade contains five maternal care species, three species whose habits are unreported and one species, *Platyphora aulica*, which has apparently lost parental care. The latter is particularly interesting in that it is distributed over the northern portion of the Brazilian shield in “cerrado” habitats resembling those occupied by species in the non-maternal care clade. Further, our tree raises doubts about present generic concepts, at least for Solanaceae-feeding taxa. This problem is best seen in the small well-resolved clade containing *Platyphora aulica*, *Platyphora amabilis* and *Proseicela vittata* in Fig. 5. Taking into consideration their non-natural grouping and the fact that a short mesothoracic horn is possessed by all members of the ingroup, but not by other *Platyphora* species (D.W. personal observation), one solution may eventually consist of transferring most, or perhaps all, short-horned, Solanaceae-feeding *Platyphora* species into the genus, *Proseicela*.

New records of maternal care in New World Chrysomelinae are beginning to reveal patterns. The first is that maternal care behavior is primarily defensive in nature. *Doryphora* mothers straddle larvae and take measures to physically confront pedestrian predators. There is no convincing evidence that mothers act to supply or direct offspring to resources, although there are indications that *Proseicela vittata* mothers are modifying resources through vein-pinching. *Proseicela* and *Platyphora* mothers closely tend, straddle and herd offspring much as observed in *Doryphora*, but aggressive challenges of nearby insects or artificial stimuli have not been observed. For the most part, mothers remain immobile and close to their larvae (e.g. the tachinid parasitoid on the *Proseicela spectabilis* larva, Fig. 3h). But, elaborate vein-pinching by *Proseicela vittata* mothers on the natal leaf just prior to and after larvaposition brings a dimension to maternal care which is apparently absent in *Doryphora* species. Presently, we do not know whether vein-pinching behavior simply deactivates plant defensive canals (Dussord 2009) or has an unknown effect on plant chemistry. Regardless, it seems the behavior is for the benefit of vulnerable offspring who exclusively feed on that part of the leaf.

As *Doryphora* species are specialist feeders on Apocynaceae it is entirely possible that adults have a defense system based on the dual sequestration of plant amyrins and lycopsamine-type alkaloids (Termonia et al. 2002). Adult *Proseicela* and *Platyphora* species associated with Solanaceae (e.g. *Platyphora microspina*), are likely to sequester only plant amyrins as precursors of saponin-defensive secretions but whether the secretions of either of these taxa are employed in defense of larvae remains unknown.

The second pattern beginning to emerge concerns choice of food plants. New World Chrysomelinae are specialist feeders on one of approximately nine different host plant families, yet all maternal care species to date are restricted to only two, Apocynaceae and Solanaceae. Clearly more documentation is desirable, but if this pattern were to continue then it will be important to look for attributes possessed by these two plant families which promote evolution of maternal defenses but which are absent in other families, such as the Malpighiaceae, a family which hosts many poorly known Chrysomelinae species in South America.

We note that all eight maternal care species in the New World occur in distinctly tropical latitudes whereas subsocial *Gonioctena* species occur from Europe (Lengerken 1939, Goidanich 1956) to Japan (Kudô and Ishibashi 1995, 1996; Kudô et al. 1995, Kudô and Hasegawa 2003). More recently, Reid et al. (2009) documented morphology and aspects of maternal care in the Australian chrysomeline beetle, *Pterodunga mirabile* Daccordi, a viviparous species associated with Proteaceae (Daccordi 2000). Female *Pterodunga mirabile* adopt a position at the base of the leaf lamina, facing toward tightly-grouped feeding larvae. Whether mothers continue to guard from the leaf petiole during resting periods as in Asian *Gonioctena sibirica* (Kudô and Ishibashi 1996) or approach or even straddle younger offspring as occurs in Neotropical care-providing species remains unclear. We learn additionally from Reid’s interesting account that once the leaf is eaten the female moves aside and larvae wander separately to leaves, while other larvae are herded so they rejoin the group.

Mafra-Neto and Jolivet (1996) proposed a tradeoff exists between cannibalism and other forms of parental investment. The rareness of cannibalism in Chrysomelinae was hypothesized to be due to other forms of costly investment in offspring, such as aggregated larvae, although it is not clear how this behavior can be seen as a cost for the parent. We would expect their argument extends to include other forms of investment such as provisioning larvae with trophic eggs. Thus *Doryphora paykulli* is not kind to their hypothesis as mothers invest heavily in both maternal care and through egg cannibalism–whether fertile or not. In contrast, the study of *Doryphora* sp. near *punctatissima* by Eberhard (1981) is friendlier to their proposal as mothers do not defend or herd offspring but larvae consume 20 to 40% of eggs. The extensive guarding by *Doryphora reticulata* mothersand thelack of egg consumption by larvae is again consistent with the idea of a tradeoff. Thus the variation in traits we observe among just three species of *Doryphora* cast some doubt on their hypothesis. Further, we have yet to observe larval cannibalism in the larviparous species of *Platyphora* and *Proseicela* associated with Solanaceae. Indeed, we suggest the nearly synchronous deposition of larvae may largely preclude cannibalism, while the staggered deposition of eggs by *Doryphora* females may facilitate the origin and maintenance of this behavior. Testing the Mafra-Neto and Jolivet (1996) hypothesis will require the study of additional species and forms of maternal investment, its synchronicity and perhaps even a search for hidden factors such as bacterial parasites (e.g. *Wolbachia* and *Spiroplasma*), well-studied manipulators of host reproduction (Werren 1997).

## Conclusion

Maternal care behavior appears limited to three genera of Neotropical Chrysomelinae and is not present in all species of these genera. *Doryphora* species are exclusively associated with lianas in the family Apocynaceae, possess a long mesosternal horn, are oviparous and reproduce at the transition from dry to wet seasons. *Proseicela* and some species currently placed within *Platyphora* are associated with Solanaceae host plants, and are live-bearing throughout the year. While extreme rareness remains an impediment to the study of most *Doryphora* species, *Proseicela* and allied species on the eastern slope of the Andes in Ecuador can be found more predictably on moderately common host plants. Large voids remain in our understanding of the natural history of both groups, including the identity and importance of predators and parasitoids and the diverse ways in which mothers may be influencing the survival of offspring.

## Supplementary Material

XML Treatment for Doryphora paykulli

XML Treatment for Doryphora reticulata

XML Treatment for Platyphora selva

XML Treatment for Platyphora microspina

XML Treatment for Proseicela vittata

XML Treatment for Proseicela bicruciata

XML Treatment for Proseicela spectabilis
